# Selective recognition and stabilization of new ligands targeting the potassium form of the human telomeric G-quadruplex DNA

**DOI:** 10.1038/srep31019

**Published:** 2016-08-11

**Authors:** Yi-Hwa Lin, Show-Mei Chuang, Pei-Ching Wu, Chun-Liang Chen, Sivakamavalli Jeyachandran, Shou-Chen Lo, Hsu-Shan Huang, Ming-Hon Hou

**Affiliations:** 1 Institute of Genomics and Bioinformatics and Institute of Life Sciences, National Chung Hsing University, Taichung 402, Taiwan; 2 Institute of Biomedical Sciences, National Chung Hsing University, Taichung 402, Taiwan; 3 Graduate Institute of Life Sciences and School of Pharmacy, National Defense Medical Center, Taipei 114, Taiwan; 4 Graduate Institute of Cancer Biology and Drug Discovery, College of Medical Science and Technology, Taipei Medical University, Taipei 110, Taiwan

## Abstract

The development of a ligand that is capable of distinguishing among the wide variety of G-quadruplex structures and targeting telomeres to treat cancer is particularly challenging. In this study, the ability of two anthraquinone telomerase inhibitors (NSC749235 and NSC764638) to target telomeric G-quadruplex DNA was probed. We found that these ligands specifically target the potassium form of telomeric G-quadruplex DNA over the DNA counterpart. The characteristic interaction with the telomeric G-quadruplex DNA and the anticancer activities of these ligands were also explored. The results of this present work emphasize our understanding of the binding selectivity of anthraquinone derivatives to G-quadruplex DNA and assists in future drug development for G-quadruplex-specific ligands.

The human genome is abundant in G-rich sequences, which are a prerequisite for the formation of G-quadruplex structures^1^,^2^. Four guanines on the same plane from each G-tract are held together via the hydrogen bonds of Hoogsteen base pairing to form a G-quartet (G4). The presence of G4s in human cells has added credence to the concept that G4s can be targets for therapeutic intervention at the single gene or poly-gene levels^3^. G-quadruplex DNA is involved in a variety of cellular activities, such as DNA replication and DNA transcription^4^,^5^. In addition, G-quadruplex structures are associated with human telomeres and involved in telomere protection^6^. Telomerase is a ribonucleoprotein complex consisting of reverse transcriptase and a RNA template; its activity is low in somatic cells and high in stem and cancer cells^7^. Experimental results demonstrated that the stability of human telomeric G-quadruplexes can prevent the unlimited elongation of telomeres by telomerase^8^,^9^. Therefore, stabilization of G-quadruplexes through ligand binding is needed to inhibit telomere elongation, which is believed to be a potential strategy for anticancer therapies^3^,^10^,^11^.

Furthermore, a variety of cations are capable of inducing G-quadruplex formation and stabilization^11^,^12^. Differences in the binding properties of potassium (K^+^) and sodium (Na^+^) ions have been discussed in terms of metal ion switches in G-quadruplexes (see Supplementary Fig. S1a)^12^. The G4 architecture has distinct features and unique structural topologies that determine the potential modes of ligand binding: tetrad-stacking, groove-binding, and loop-binding^13^. Every G-tetrad ends in a G4 structure that provides a chemically distinct environment that influences its interactions with small molecules. Appropriate ligands could stabilize G4s, and the resulting complexes help to maintain the integrity of telomeres, transcription or translation; depending on the nature of the quadruplex target site, these effects are reflected in human cancer genes (proto-oncogenes), such as c-myc and c-kit^3^. A large number of small molecules have been reported to be G4-binding ligands, including porphyrins, quinacridones, anthraquinones, phenanthrolines, substituted triazines and acridines, which have previously been shown to bind to and stabilize the quadruplex structure of telomeric DNA^14^,^15^. These ligands are capable of interactive stacking with the G-tetrads through a number of distinct mechanisms involving intercalation, end pasting, “sandwich”-type stacking, groove binding, or nonspecific external events involving charge neutralization. Several cyclic and acyclic analogues have been reported, some of which show potent biological activity^12^.

Heteroaromatic compounds with large flat surfaces interact with the terminal G-quartet in a typical quadruplex structure. Similarly, cyclic poly-oxazole and the natural compound telomestatin interact in the same manner, as do acyclic compounds, such as pyridostatin and phenyl- and pyridylbis-oxazoles, which tend to be characterized by a crescent shape^16^,^17^,^18^,^19^,^20^; these compounds all selectively target G4s. A more general requirement is that most G4-binding ligands should possess side-chains that terminate with a cationic charge^21^. As recently demonstrated, anthraquinone derivatives (AQs) exemplify an interesting scaffold for developing selective and multifunctional G4 ligands^15^,^22^,^23^. Percivalle *et al*. reported the differences in the binding properties of aryl ethynyl anthraquinones to G-quadruplex DNA^24^; these findings emphasize the possibility that new compounds could be promising candidates for the development of a new generation of multifunctional G4-interacting ligands.

In this study, two chemically synthesized anthrax[1,2-d]imidazole-6,11-dione tetracyclic analogues (NSC749235 and NSC764638) as novel telomerase inhibitors, which are addition of a fourth planar aromatic system to a tricyclic chromophore, were used to explore their specificity on the telomeric G-quadruplexes (Fig. 1a, see Supplementary Figs S1b and S2). This present study investigates the mechanism of tetracyclic anthraquinone derivatives (NSC749235 and NSC764638) binding to G4s using biophysical analyses, such as a FRET melting assay, UV-vis absorption spectrophotometry, SPR analysis, and CD spectroscopy. The cytotoxicity of the two anthraquinone derivatives against cancer cell lines was also evaluated. We envisage that the tetracyclic anthraquinone derivatives will specifically stabilize the potassium form of human telomeric G-quadruplex DNA and, to a lesser degree, other forms of G-quadruplex DNAs. The present study will provide insights into the development of specific drugs targeting G-quadruplex DNA.

## Results and Discussion

### The thermodynamic stability and conformational analyses of the tetracyclic anthraquinone derivatives binding to telomeric G-quadruplex DNAs

**Figure 1:**
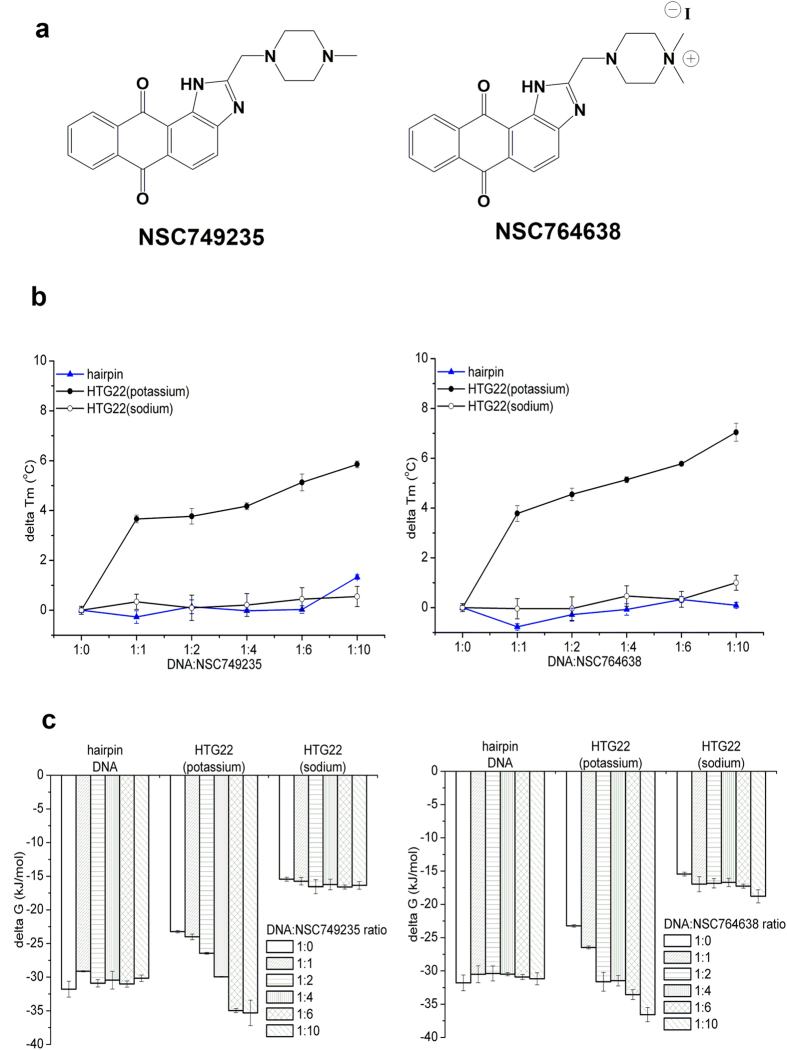
(**a**) Chemical structures of NSC749235 (left) and NSC764638 (right). (**b**) Effects of NSC749235 (left) and NSC764638 (right) at various concentration ratios on the delta Tm values of hairpin DNA, HTG22 (potassium), and HTG22 (sodium). (**c**) Values of delta G (kJ/mol) of hairpin DNA, HTG22 (potassium), and HTG22 (sodium) were incubated in the presence of NSC749235 (left) and NSC764638 (right) at various concentrations.

**Figure 2:**
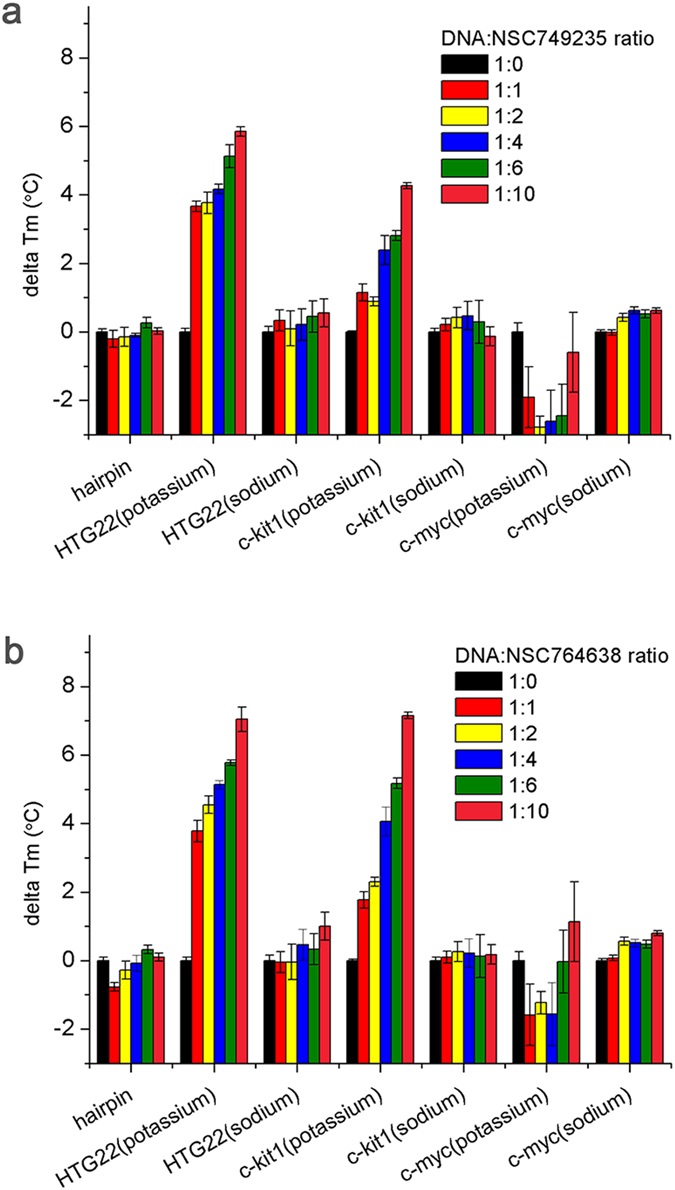
Values of delta Tm of hairpin, HTG22 (potassium), HTG22 (sodium), c-kit 1 (potassium), c-kit 1 (sodium), c-myc (potassium) and c-myc (sodium) forms of DNA were incubated with NSC749235 (**a**) and NSC764638 (**b**) at various concentrations.

Human telomeric sequences fold into a G-quadruplex and undergo conformational changes that depend on the presence of cations, such as K^+^ and Na^+ ^25,26,27,28. To evaluate the thermodynamic stability and affinity of telomeric G-quadruplex DNA (K^+^ and Na^+^ models) binding to the synthesized two tetracyclic anthraquinone derivatives, NSC749235 and NSC764638, the T_m_ value was calculated using a fluorescence resonance energy transfer (FRET) melting assay (see Supplementary Fig. S3a and b). We also synthesized a hairpin DNA duplex as a negative control (see Supplementary Fig. S3c). The DNA sequences and their extinction coefficients are shown in Supplementary Table S1. The stabilization effects of these two anthraquinone derivatives on DNA were measured from the ∆T_m_ values when the potassium form of telomeric G-quadruplex DNA was treated with NSC749235 and NSC764638; these values are shown in Tables 1 and 2, and the concentration-dependent melting curves are shown in Fig. 1b. The stabilization of potassium-containing telomeric G-quadruplex DNA by NSC749235 and NSC764638 led to ∆T_m_ values ranging from 3.66 to 8.04 °C. In contrast, the T_m_ value of hairpin DNA duplex was not affected by presence of NSC749235 and NSC764638 (Tables 1 and 2). As shown in Table 2, NSC764638 (20 μM), with a ∆T_m_ value of 8.04 °C, was better able to stabilize the potassium form of telomeric G-quadruplex DNA. Our results were consistent with the existing reports indicating that the salt form of the compound would better stabilize G-quadruplex DNA^7^,^8^,^9^. For the sodium form of the telomeric G-quadruplex, 20 μM of these two anthraquinone derivatives merely increased the T_m_ values from 0.55 to 1.00 °C (Tables 1 and 2). The extent of G-quadruplex DNA formation in the presence of the compounds is indicated by the ΔG value. The ΔG values for the potassium form increased upon the addition of the two compounds (Fig. 1c); however, the ΔG values for the sodium form were not affected by the compounds (see Supplementary Tables S2 and S3). Therefore, the stabilizing effects of the two anthraquinone derivatives (NSC749235 and NSC764638) on the potassium form in terms of melting G-quadruplex DNA is much greater than their effects on the sodium form. As shown in Fig. 2, we showed that there were no significant changes in the ∆Tm values of the G-quadruplex in the c-myc (Na^+^ and K^+^ form) and c-kit 1 (Na^+^ form) upon NSC749235 and NSC764638 binding. These two anthraquinone derivatives were able to increase the ∆Tm values of the potassium form of the c-kit 1 G-quadruplex; however, their stabilizing effects were lower than the effects on the potassium form of telomeric G-quadruplex DNA at various concentrations. Based on these results, we suggest that the ligands showed higher selectivity for the potassium form of the telomeric G-quadruplex than for the sodium form of the G-quadruplex or the oncogene promoter G-quadruplexes.

**Figure 3:**
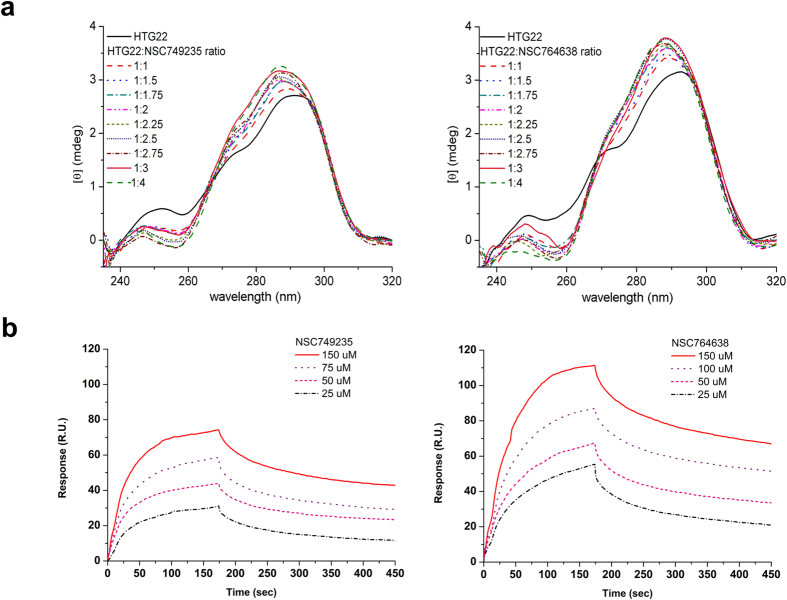
(**a**) CD spectra of HTG22 (potassium) incubated in the presence of NSC749235 (left) and NSC764638 (right) at various concentrations. CD titration of HTG22 (potassium) tested against NSC749235 and NSC764638 with varying CD intensities at 285 nm with different molar ratios. The two solid lines represent the initial binding curve of the compound to HTG22 (potassium) and the curve that reaches the plateau. (**b**) Sensorgrams of the interaction between the immobilized HTG22 (potassium) and NSC749235 (left) or NSC764638 (right) at various concentrations.

Circular dichroism (CD) spectroscopy was employed to determine the conformation of the G-quadruplex in the presence of the anthraquinone derivatives. Similar to previous studies, a CD spectrum was used to characterize the conformations of the potassium (K^+^) and sodium (Na^+^) forms of the G-quadruplex^29^,^30^,^31^,^32^,^33^. A human telomeric sequence (HTG22) formed a typical antiparallel quadruplex DNA structure in the presence of Na^+^, with a large positive band at 295 nm and a negative band at 265 nm in the CD spectra. The CD spectra of HTG22 in the presence of K^+^ ions exhibited a large positive band at 290 nm, a small positive band at 265 nm, and a negative band at 235 nm, which suggested that HTG22 might exist as a hybrid-type of quadruplex DNA containing parallel and antiparallel structures^34^,^35^. Upon the addition of the compounds (NSC749235 and NSC764638) to HTG22 in buffer containing K^+^ ion, the CD spectra changed; the small positive band at 265 nm disappeared, indicating the possible destruction of the parallel structure in the G-quadruplexes. In contrast, the positive band at approximately 290 nm increased obviously, and a negative band at approximately 260 nm appeared, which suggests the formation of an antiparallel structure (Fig. 3a). CD studies were also carried out to examine the effects of compounds on the G-quadruplex (sodium from), hairpin DNA, and promoter G-quadruplexes (see Supplementary Fig. S4). However, upon the addition of NSC749235 and NSC764638 to these DNAs, the CD spectra showed relatively little change in the DNA structure upon the drug binding.

These CD observations are consistent with the Tm results, suggesting that these two anthraquinone derivatives prefer the human telomeric potassium form of G-quadruplex over the DNA counterpart. Additionally, the stoichiometry of the binding of the anthraquinone derivative to the G-quadruplex was determined from CD spectra. When NSC749235 and NSC764638 were titrated against HTG22 in the presence of K^+^ ions, the band at 285 nm gradually increased until the ratio of NSC749235/NSC764638 to HTG22 was equal to 2:1. The changes in ellipticity at 285 nm as the molar ratio were varied (NSC749235/HTG22 and NSC764638/HTG22). The CD spectra and the breakpoints suggested the formation of a 2:1 NSC749235/NSC764638−HTG22 complex.

### DNA binding affinity analyses of the tetracyclic anthraquinone derivatives to the potassium form of G-quadruplex

To characterize the binding affinity of NSC749235 and NSC764638 for the potassium form of G-quadruplex, the compounds were allowed to interact with biotin-labeled HTG22 that formed G-quadruplexes in the presence of potassium ion at various concentrations, and the results were monitored by SPR. In Fig. 3b, the association between the G-quadruplex and anthraquinone derivatives is shown by an increase in the RU values, whereas the dissociation of these two species is indicated by a decrease in the same trace. The SPR sensorgram indicated that the DNA-binding capacity of NSC764638 to HTG22 (~111 RU) was higher than the binding capacity of NSC749235 to this G-quadruplex (~74 RU) at same concentration. The binding of anthraquinone derivatives to hairpin DNA was carried out by SPR, and the results showed that the binding capacity of anthraquinone derivatives to hairpin DNA (~39 RU for NSC764638 and ~19 RU for NSC749235) was lower than those to HTG22 (see Supplementary Fig. S5).

Kinetic experiments were performed using measurements of the binding parameters for the anthraquinone derivatives and their target G-quadruplex (Table 3). The kinetic constants of association (k_a_ in M^−1^s^−1^) and dissociation (k_d_ in s^−1^) for the anthraquinone derivatives binding were calculated from the association and dissociation phases of the SPR traces, respectively. HTG22 has two sites for compound binding. The k_a1_, k_d1_ and k_a2_, k_d2_ values that contribute to the respective association constants (K_a1_ and K_a2_) of the two binding modes were obtained for the HTG22-compound interactions. Compared with NSC749235, NSC764638 was shown to have higher k_a1_ and k_a2_ values. For the dissociation rate constants, the k_d1_ values of NSC764638 and NSC749235 were essentially the same. Compared with NSC764638, NSC749235 had a higher k_d2_. The association constants (K_a_) were calculated as k_a_/k_d_ (M^−1^) and are listed in Table 3. NSC764638 showed higher K_a1_ and K_a2_ values, which were ~2-fold and 4-fold larger than the K_a1_ and K_a2_ of NSC749235, respectively, indicating preferential binding of HTG22 to the sodium form. In addition, NSC764638 had a higher K_a2_ value that was ~1.5-fold greater than the K_a1_ of NSC764638, which suggests that the binding of two NSC764638 molecules to HTG may exhibit negative cooperativity. The K_a_ of NSC749235 and NSC764638 with hairpin DNA were also calculated to be 6 × 10^4^ M^−1^ and 4 × 10^4^ M^−1^, respectively, that were ~3 and 7-fold lower than the K_a_ of the anthraquinone derivatives with the potassium form of HTG22.

### Molecular modeling of the tetracyclic anthraquinone derivatives interacting with the potassium form of G-quadruplex based on the physical experimental results

**Figure 4:**
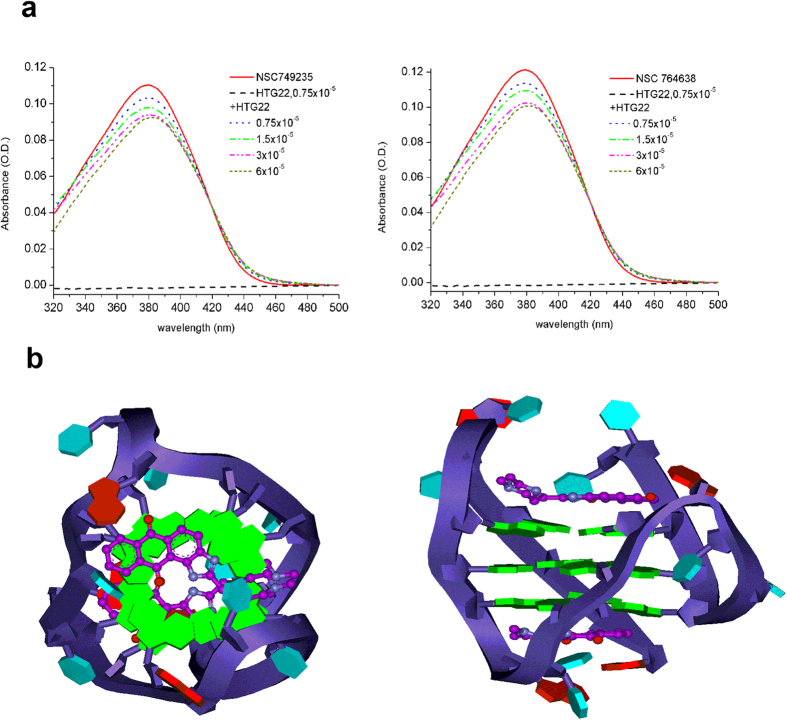
(**a**) UV spectra of NSC749235 (left) and NSC764638 (right) incubated in the presence of HTG22 (potassium form) at various concentrations. (**b**) Drawings of the model of NSC749235 (left) bound to HTG22 (potassium form) viewed from the 5′ end of the quadruplex looking down the helical axis with the phosphate sugar backbone drawn as a blue ribbon showing the 5′-to-3′ directionality. NSC749235 are purple, guanines are green, thymines are cyan, and adenines are red. Side view of the model of NSC749235 (right) bound to HTG22 (potassium form). Guanines are green, and NSC749235 is purple. Molecular dynamics simulation has been used to construct the proposed coordination geometry using the Discovery Studio program with the 2MB3 PDB file.

Previous reports suggested that the electrochemical properties of anthraquinone could be detected using absorption spectra^36^. The maximum absorption peak of the lowest energy transition for quinones is due to the energy difference between the highest occupied molecular orbital and the lowest unoccupied molecular orbital (HOMO and LUMO, respectively). For NSC749235 and NSC764638, the energy of the n–π^*^ transitions between 375 nm and 425 nm was determined. Figure 4a shows the absorption spectra of the compounds (15 μM) with 0–60 μM HTG22 DNA. When bound to HTG22 DNA, both compounds exhibited moderate hypochromicity and insignificant red shifts, with the maxima at 380 nm for NSC749235 (16.41%, 3 nm) and at 380 nm for NSC764638 (16.89%, 1 nm). An isosbestic spectral change was observed in both spectra near 420 nm. The overall spectral changes, including hypochromicity, an insignificant red shift and an unclear isosbestic point, induced by the binding of planar polyaromatic drugs to DNA suggests that non-intercalative association is the major binding mode^37^. The hypochromicity in the absorption spectra is mostly attributed to the interaction between the electronic states of the chromophore compound and those of the DNA bases. In contrast, the red shift is associated with a decrease in the energy gap between the HOMO and LUMO when the drug compound binds to DNA. Moreover, the unclear isosbestic points in NSC749235 and NSC764638 indicate the presence of more than two spectroscopically distinct chromophores in the solution^38^,^39^,^40^. In other words, the major binding mode of these compounds with DNA may be stacking onto the 5′ or 3′-end G-quartet. We performed molecular modelling of the HTG22 intramolecular G-quadruplex loop isomer model to further investigate the mode of binding. Neither NMR nor X-ray crystallographic information is available for hybrid type HTG22 complexed with tetracyclic anthraquinone derivative; thus, a model was built from the solution structure of a known, closely related human intramolecular telomeric G-quadruplex DNA complexed with telomestatin derivative (PDB code: 2MB3)^41^. The UV titration and CD spectra results indicated that the compounds bind to HTG22 through a stacking interaction onto the 5′ or 3′-end G-quartet with a molar ratio of 2:1 via the sandwich model (Fig. 4b). The π-π stacking possibly stabilized the binding of NSC749235 to the G-quartet at the two ends of the G-tetrad core. An electrostatic interaction might be formed between the piperazine ring N1 atom and the phosphate groups of G11 and G17 at the 5′ and 3′-ends of the G-quartet, respectively (Fig. 4b).

### Evaluation of *in vitro* cytotoxicity of the tetracyclic anthraquinone derivatives against human cancer cell lines

The *in vitro* cytotoxicity of the anthraquinone derivatives against human cancer cell lines, a cervical adenocarcinoma cell line (HeLa) and a non-small cell lung carcinoma cell line (A549) was investigated using a tetrazolium-based (MTS) colorimetric assay and compared with those of Daunorubicin (an anticancer drug used widely in the clinic) as a positive control (Table 4). The resulting *in vitro* cytotoxic activity values are expressed as the IC_50_, the concentration of compound that inhibits the cell survival by 50% compared with that of control untreated cells after 24 and 48 hours. NSC794235 exists inhibitory activity against the tested cancer cell lines, with IC_50_ values ranging from 5.54 to 14.54 μM. Our results indicate that the HeLa cell line is much more sensitive to the NSC794235 than the A549 cell line (Table 4). In contrast, the salt form compound, NSC764638, presented relatively lower inhibitory activity (IC_50_ greater than 50 μM). Due to the presence of a positively charged group in its chemical structure, we determined the permeability of the anthraquinone derivative (NSC764638) on A549 cells, and demonstrated that NSC764638 didn’t penetrate the membrane of the A549 cells (see Supplementary Fig. S6), which could be the reason behind the decreased inhibitory activity, as reflected in the IC_50_ value^42^. This selectivity indicates that NSC749235 could be a leading compound for designing drugs against cancer.

## Conclusions

We report the synthesis of two geometrically flexible tetracyclic anthraquinone derivatives as the basis of a novel class of telomerase inhibitors that exhibits highly specific potential for stabilizing the potassium form of human telomeric G-quadruplex DNA over the analogous sodium form. The mechanisms for the binding of the tetracyclic anthraquinone analogues to human telomeric G-quadruplex DNA and the cytotoxicity of the compounds against cancer cell lines were also evaluated. The cytotoxicity results indicated that NSC794235 at μM levels can inhibit cancer cell growth. The studies suggest that these or related compounds may act as a small molecule scaffold and be promising candidates for the structure-based design and development of G-quadruplex-specific ligands.

## Methods

### G-quadruplex DNAs preparation and synthesis of chemical compounds

The schematic diagram of the potassium and sodium of G-quadruples was shown in Supplementary Fig. S1a. The oligonucleotides of G-quadruplex DNA sequences were purchased from Genomics (New Taipei city, Taiwan). The potassium and sodium forms of G-quadruplexes were prepared in the presence of 100 mM NaCl and 100 mM KCl, respectively, with brief heating of the DNA mixture to 95 °C, followed by slow cooling to room temperature (−0.5 °C/min). NSC749235 and NSC764638 were synthesized and provided by Dr. Hsu-Shan Huang. The details of the synthesis and chemical characterization of these two compounds have been reported in a previous study^43^. Stock solutions of the two derivatives (10 mM) were prepared using DMSO (10%).

### Telomere repeat amplification protocol (TRAP assay)

Telomerase activity was performed according to the previous study^43^. Briefly, the oligonucleotides generated by the action of telomerase on a TS primer by PCR amplification, and the PCR products were resolved by 10% polyacrylamide gel electrophoresis and stained with SYBER Green.

### Fluorescence resonance energy transfer (FRET) assay

All the oligonucleotides of G-quadruplex DNA and their fluorescent conjugates were initially dissolved in purified water to form a 100 μM stock solution; further dilutions were carried out in the relevant buffer. The ability of the compounds to stabilize G-quadruplex DNA the fluorophores FAM at the 5′-end as a donor and TAMRA at the 3′-end as an acceptor was investigated using a FRET assay that was performed in triplicate using a real-time PCR apparatus (Rotor Gene 3000, CORBETT RESEARCH, Sydney, Australia)^44^. Fluorescence readings with excitation at 470 and detection at 510 nm were recorded at intervals of 1 °C over the range 30–95 °C; a constant temperature was maintained for 10 min prior to each reading to ensure a stable value.

### Determination of thermodynamics parameters

UV absorbance versus temperature profiles of hairpin DNA duplex was generated by measuring the sample absorption (in O.D.) at 260 nm using a JASCO UV/VIS spectrophotometer (JASCO, Tokyo, Japan). The sample cell was equipped with a Peltier-type cell holder (EHC-441), and the temperature was regulated with a programmer (JASCO TPU-436). The concentration of the duplex DNA in each sample was 3 μM in a 20 mM cacodylic acid-buffered solution with 100 mM KCl at pH 7.3. The experiments were performed by increasing the temperature at a rate of 1 °C/min from 5 to 95 °C, and the temperature was recorded every 0.5 min. The data set of each melting curve was normalized to minimize variations in each experiment because T_m_ is independent of the DNA concentration^45^. To obtain van’t Hoff transition enthalpies, the UV melting curves were evaluated; thus, the experimental absorbance versus temperature curve could be converted into a curve of melted fraction versus temperature. Plots displaying the melted fractions in single strands (f) versus temperature (T) were calculated by fitting the melting profile to a two-state transition model. The T_m_ values were evaluated directly from the temperature at f = 0.5. The equilibrium constants and thermodynamic parameters of DNA interacting with and without compounds were estimated using the melting profiles according to previous methods^46^. Briefly, the equilibrium constant, K, at a given temperature, T, was calculated using the following equation: K = (1 – f)/[(C_T_/n)(n^−1^)fn], where f is the melted fraction in single strands, n is the molecularity of the reaction, and C_T_ is the total concentration of strands. The enthalpy change, ΔH, was determined from the temperature dependence of the equilibrium constant, K. ΔH was calculated from the slope of a ln K_a_ vs. 1/T plot according to the equation, ln K_a_ = −ΔH/RT + ΔS/R, where ΔS, the entropy change, was obtained from the ordinate at the origin of the fitted line. The Gibb’s free energy change, ΔG, at 25 °C was calculated from the following relationship: ΔG = ΔH – TΔS.

### SPR binding analysis

The affinity, association and dissociation between the chosen anthraquinone derivatives (NSC749235 and NSC764638) and telomeric G-quadruplex DNAs or hairpin DNA were measured in a BIAcore 3000A SPR instrument (Pharmacia, Uppsala, Sweden) with a SensorChip SA5 (Pharmacia) that monitored changes in the refractive index at the surface of the sensor chip. These changes are generally assumed to be proportional to the mass of the molecules bound to the chip and are recorded in resonance units (RUs)^47^. 5′-biotin-labeled DNA purified by polyacrylamide gel electrophoresis (PAGE) was used in the SPR experiments. The SPR binding constants were calculated using a bivalent ligand model as previously described^47^. Sensorgrams for the interactions between the DNA and the drug were analyzed using the BIA evaluation software (version 3).

### CD spectroscopy

CD experiments were performed on a JASCO J-810 spectropolarimeter (JASCO, MD, USA). A quartz cuvette with a 1 cm path length was used for spectra, which were recorded from 240 to 320 nm at a 2 nm bandwidth with a response time of 0.5 s. The scanning speed was 50 nm min^−1^. The reported spectrum of each sample represents the average of three scans. CD titration was performed at a fixed G-quadruplex or oligomer concentration (5 μM) with various concentrations of NSC749235 and NSC764638 in the corresponding Tris-HCl buffer. After each addition of the compound, the reaction was stirred and allowed to equilibrate for at least 30 min (until no elliptic changes were observed), and the CD spectrum was collected. A background CD spectrum of the corresponding buffer was subtracted from the average scan for each sample^45^.

### UV-vis absorbance measurements

Absorption spectra were recorded on a JASCO UV/VIS spectrophotometer (JASCO, Tokyo, Japan). A quartz cuvette with a 1 cm path length was used for spectra recorded from 320 to 500 nm. The scanning speed was 10 nm min^−1^. All titration experiments were performed at a fixed concentration (15 μM) of NSC749235 and NSC764638 with various G-quadruplex concentrations in the corresponding Tris-HCl buffer. After each addition of the compound, the reaction mixture was stirred and allowed to equilibrate for at least 30 min.

### Molecular modeling

Molecular modeling was performed on a Dell Precision workstation. In this study, we used the solution structure of a hybrid type of human telomeric DNA complexed with a telomestatin derivative, the macrocyclic hexaoxazole L2H2-6M(2)OTD, (2MB3) as a template to construct a model structure of human telomeric DNA complexed with NSC749235 using the Accelrys Discovery Studio 2.5 (DS) program. Using the human telomeric DNA-telomestatin derivative NMR structure, we constructed NSC749235 and human telomeric DNA complex for further refinement using the procedure implemented in the Accelrys Discovery Studio 2.5 (DS) program^48^. The CHARMM force field was used and the quality of the model geometry was evaluated by the r.m.s. derivation of the bond length and bond angle.

### Cell culture and cell viability assay

The human cervical cancer cell line (HeLa) and the human alveolar epithelial carcinoma cell line (A549) were provided by the American Type Culture Collection (Rockville, MD). Cellular proliferations were evaluated using the colorimetric MTS assay (CellTiter, Promega, WI, USA). Briefly, cells were seeded into 96-well plates at a density of 5 × 10^3^ cells/well and incubated for 24 h. After then, the medium was removed and replaced with different concentration of NSC749235, NSC764638 and daunorubicin in fresh medium. After an additional 24 h and 48 h, the MTS solution was added and incubated at 37 °C for an additional 1 h. The optical density was measured at 490 nm in an ELISA reader. At least three independent experiments were performed to obtain the results for a statistical analysis.

### Cellular permeability assay

The human alveolar epithelial carcinoma cell line (A549) was used to evaluate cellular permeability of NSC749235 and NSC764638. Briefly, A549 cells were seeded into 6 cm wells and incubated for 24 h. After then, the medium was removed and replaced with 5 μM of NSC749235 and NSC764638 in fresh medium. After an additional 6 h, followed by washing three times with PBS. Cells were detected by red fluorescence and phase contrast microscope.

## Additional Information

**How to cite this article**: Lin, Y.-H. *et al*. Selective recognition and stabilization of new ligands targeting the potassium form of the human telomeric G-quadruplex DNA. *Sci. Rep.* **6**, 31019; doi: 10.1038/srep31019 (2016).

## Supplementary Material

Supplementary Information

## Figures and Tables

**Table 1:** Values of delta Tm of DNAs incubated with NSC749235 at various concentrations.

Delta Tm (^o^C)	NSC749235		
DNA	Hairpin	Human telomere (potassium)	Human telomere (sodium)
DNA:drug ratio			
1:0	0	0	0
1:1	−0.20	3.66	0.34
1:2	−0.15	3.77	0.10
1:4	−0.09	4.09	0.21
1:6	0.27	4.49	0.45
1:10	0.03	5.85	0.55

**Table 2:** Values of delta Tm of DNAs incubated with NSC764638 at various concentrations.

Delta Tm (°C)	NSC764638		
DNA	Hairpin	Human telomere (potassium)	Human telomere (sodium)
DNA:drug ratio			
1:0	0	0	0
1:1	−0.77	4.63	−0.04
1:2	−0.28	5.35	−0.04
1:4	−0.07	6.20	0.46
1:6	0.32	6.52	0.33
1:10	0.10	8.04	1.00

**Table 3:** Numerical k_a1_, k_a2_, k_d1_, k_d2_, K_a1_, and K_a2_ values obtained from the kinetic analysis of the SPR experiments to examine the binding of NSC749235 and NSC764638 to HTG22 (potassium).

Compound	DNA	k_a1_ (M^−1^sec^−1^)	k_a2_ (M^−1^sec^−1^)	k_d1_ (sec^−1^)	k_d2_ (sec^−1^)	K_a1_ (M^−1^)	K_a2_ (M^−1^)
NSC749235	HTG22 (potassium)	0.29 × 10^3^	0.30 × 10^3^	1.77 × 10^3^	1.75 × 10^3^	1.65 × 10^5^	1.69 × 10^5^
NSC764638	HTG22 (potassium)	0.48 × 10^3^	0.46 × 10^3^	1.70 × 10^3^	1.20 × 10^3^	2.82 × 10^5^	3.84 × 10^5^

**Table 4:** Effects of NSC749235, NSC764638, and DNR* on the growth of two cancer cell lines after 24 and 48 h, as indicated by the IC_50_ a values (μM).

	24 h			48 h		
Cell line type	NSC749235	NSC764638	DNR*	NSC749235	NSC764638	DNR*
HeLa	14.54 ± 0.54	>50	1.58 ± 0.11	5.54 ± 0.27	>50	0.46 ± 0.06
A549	17.5 ± 0.13	>50	1.05 ± 0.13	12.35 ± 0.68	>50	0.65 ± 0.02

^*^DNR represents Daunorubicin.

^a^IC_50_ indicates the concentrations that inhibited cell growth by 50%.
