# Supplementary material for: Selective recognition and stabilization of new ligands targeting the potassium form of the human telomeric G-quadruplex DNA
Source: Sci Rep. 2016 Aug 11;6:31019. doi: 10.1038/srep31019 (PMC4980623; doi:10.1038/srep31019)
Supplement: Supplementary Information [file srep31019-s1.pdf]

Selective recognition and stabilization of new ligands targeting the potassium form of the human telomeric G-quadruplex DNA

Yi-Hwa Lin[1,+], Show-Mei Chuang[2,+], Pei-Ching Wu[1,+], Chun-Liang Chen[3], Sivakamavalli Jeyachandran[1], Shou-Chen Lo[1], Hsu-Shan Huang [3,4,*], and Ming-Hon Hou[1,*]

[1]Institute of Genomics and Bioinformatics and Institute of Life Sciences, National Chung Hsing University, Taichung 402, Taiwan

[2]Institute of Biomedical Sciences, National Chung Hsing University, Taichung 402, Taiwan

[3]Graduate Institute of Life Sciences and School of Pharmacy, National Defense Medical Center, Taipei 114, Taiwan

[4]Graduate Institute of Cancer Biology and Drug Discovery, College of Medical Science and Technology, Taipei Medical University, Taipei 110, Taiwan

[*]corresponding. mhho@nchu.edu.tw; or huanghs99@tmu.edu.tw

[+]these authors contributed equally to this work

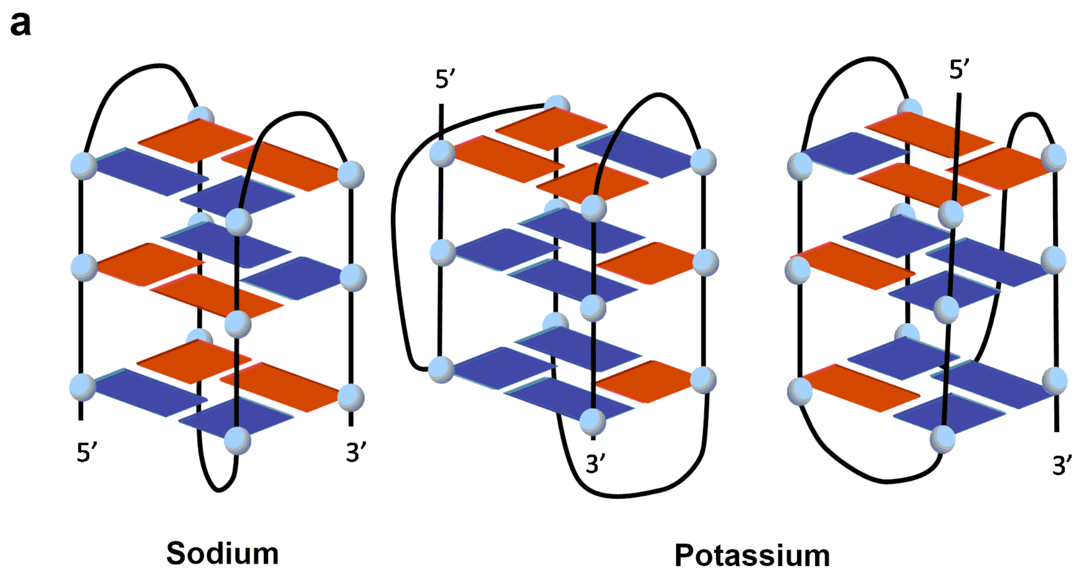

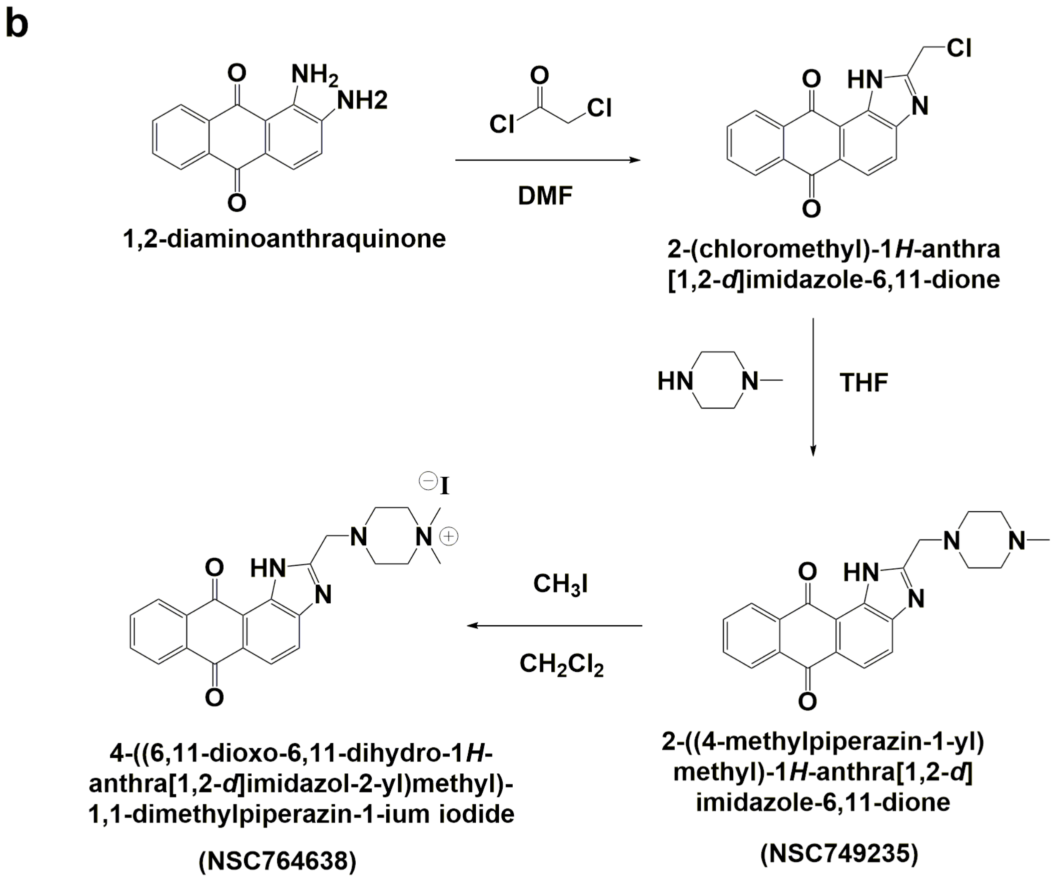

Figure S1. (a) Schematic diagram of G-quadruplex DNA. (b) Scheme of synthesizing compounds (NSC749235 and NSC764638).

TRAP assay

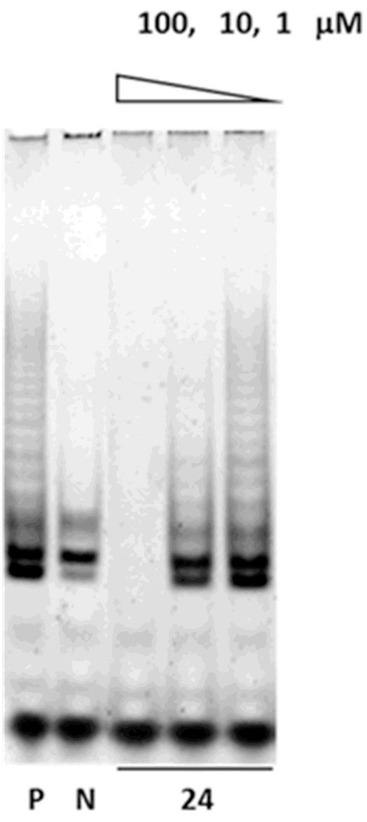

Figure S2. Telomere repeat amplification protocol assay results for compound NSC749235, showing the oligonucleotides generated by the action of telomerase on a TS primer by PCR amplification. The lowest band is an internal control band. P represents the positive control, N is the negative control, and 24 indicate the telomere sequence. TRAP assay showed that NSC749235 is able to inhibit the activity of telomerase for the concentration of compound up to 100 uM, indicating that NSC749235 can be used as a novel telomerase inhibitor.

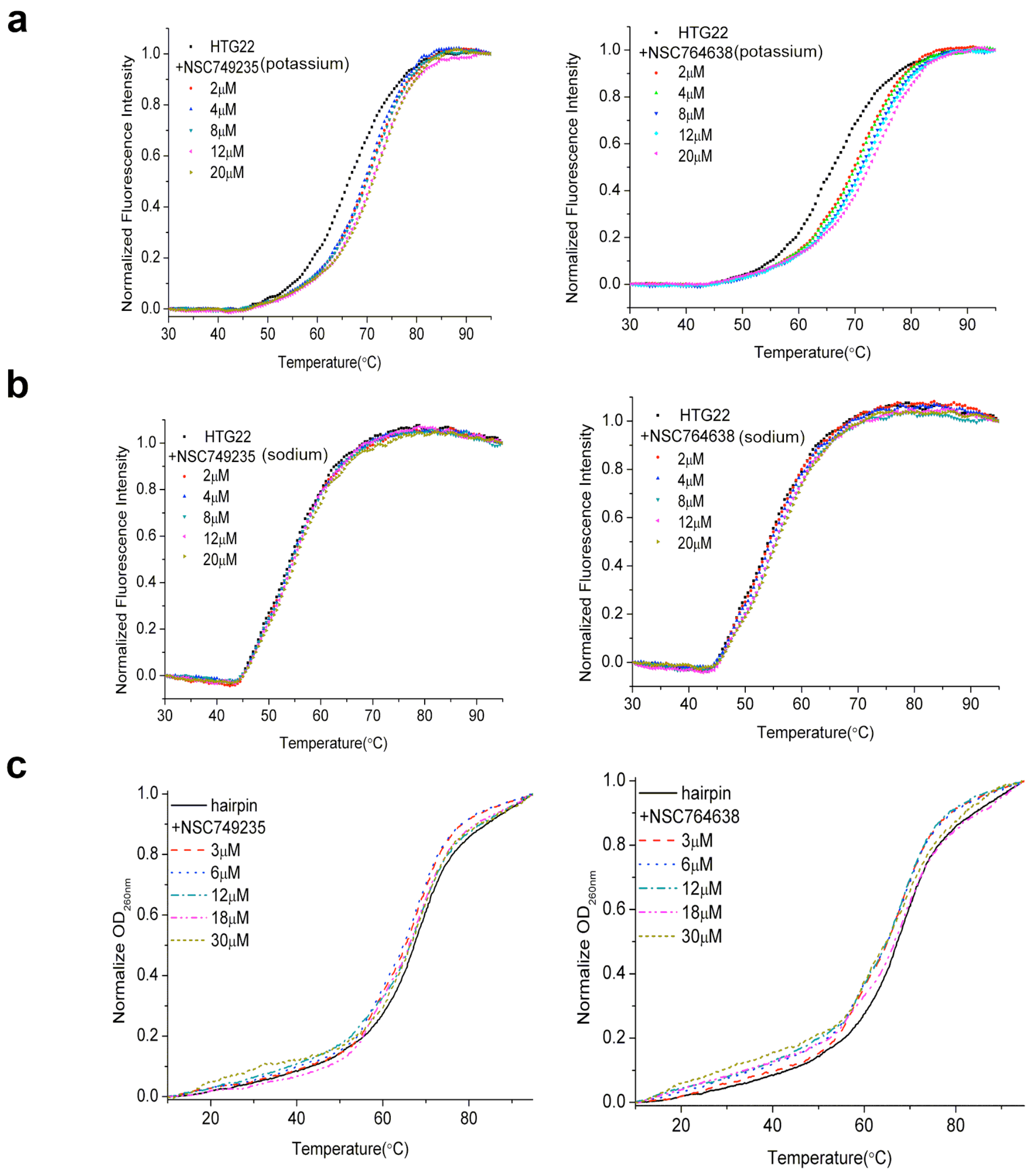

Figure S3. Melting curves of DNA incubated with NSC749235 (left) and NSC764638 (right) at various concentrations. (a) HTG22 (potassium) incubated with NSC749235 and NSC764638; (b) HTG22 (sodium) with NSC749235 and NSC764638; and (c) hairpin DNA with NSC749235 and NSC764638.

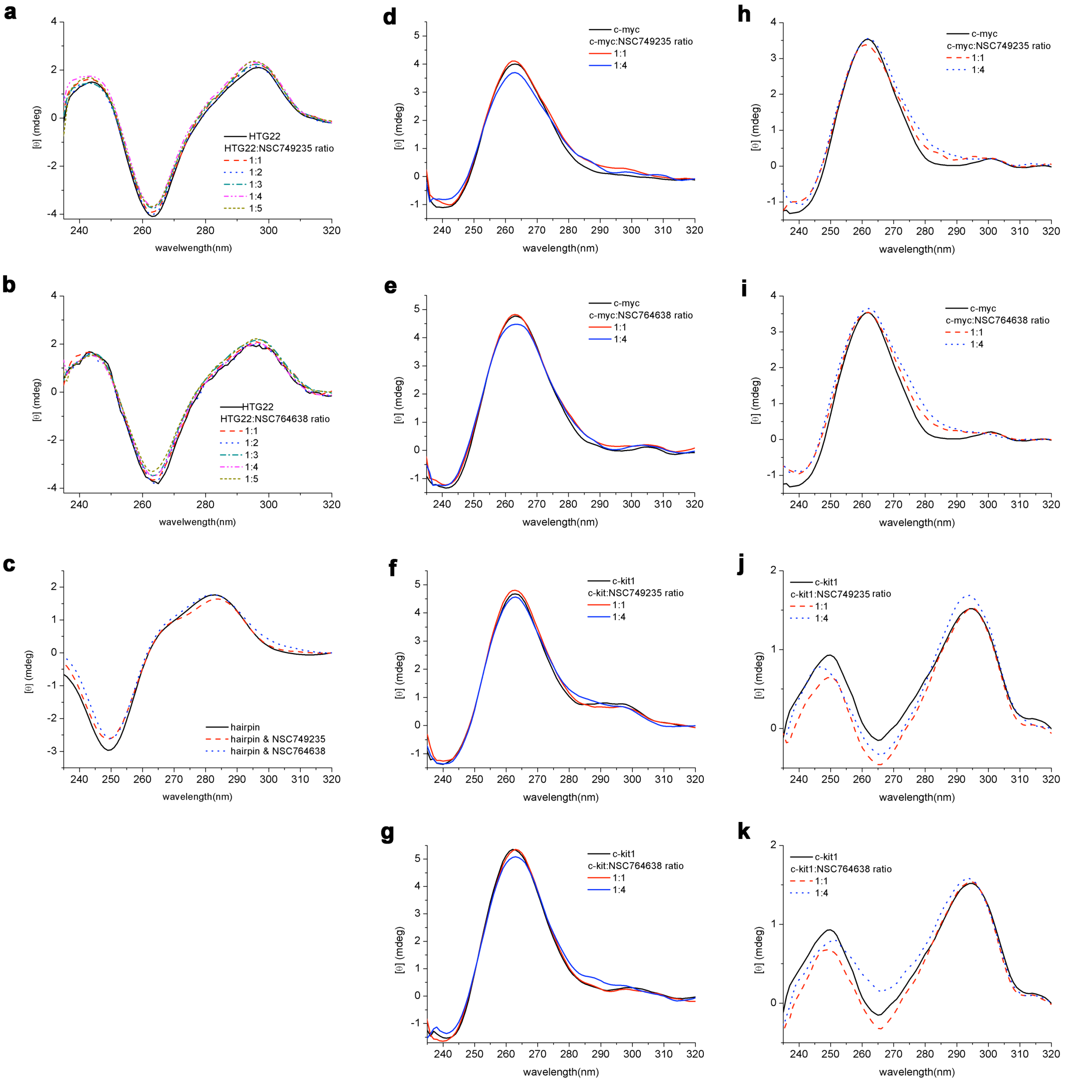

Figure S4. CD spectra of HTG22 (sodium form) incubated with compound NSC749235 (a) and NSC764638 (b) at various concentrations. (c) CD spectra of hairpin DNA incubated with NSC749235 and NSC764638. CD spectra of c-myc and c-kit (d-g, potassium from; h-k, sodium from) incubated with compound NSC749235 or NSC764638 at various concentrations.

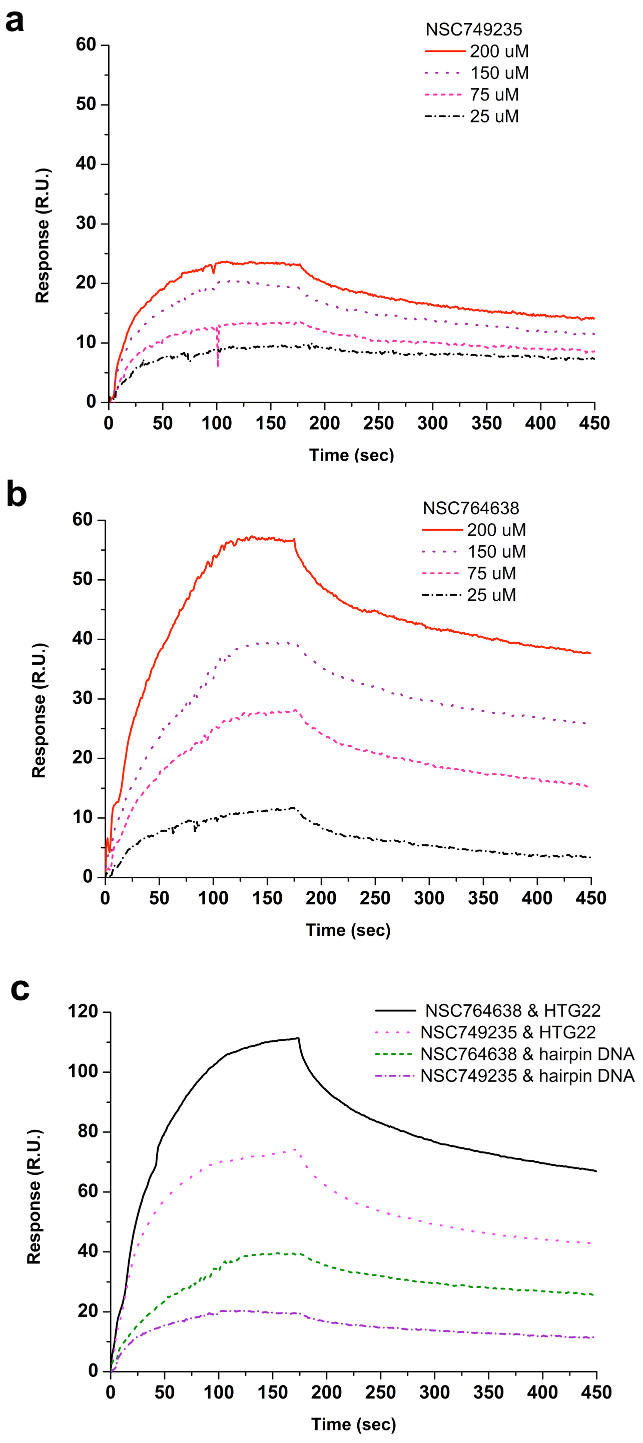

Figure S5. Sensorgrams of the interaction between the immobilized hairpin DNA and NSC749235 (a) or NSC764638 (b) at various concentrations. (c) Binding capacity sensorgram of anthraquinone derivatives (150 uM) bound to HTG22 or hairpin DNA.

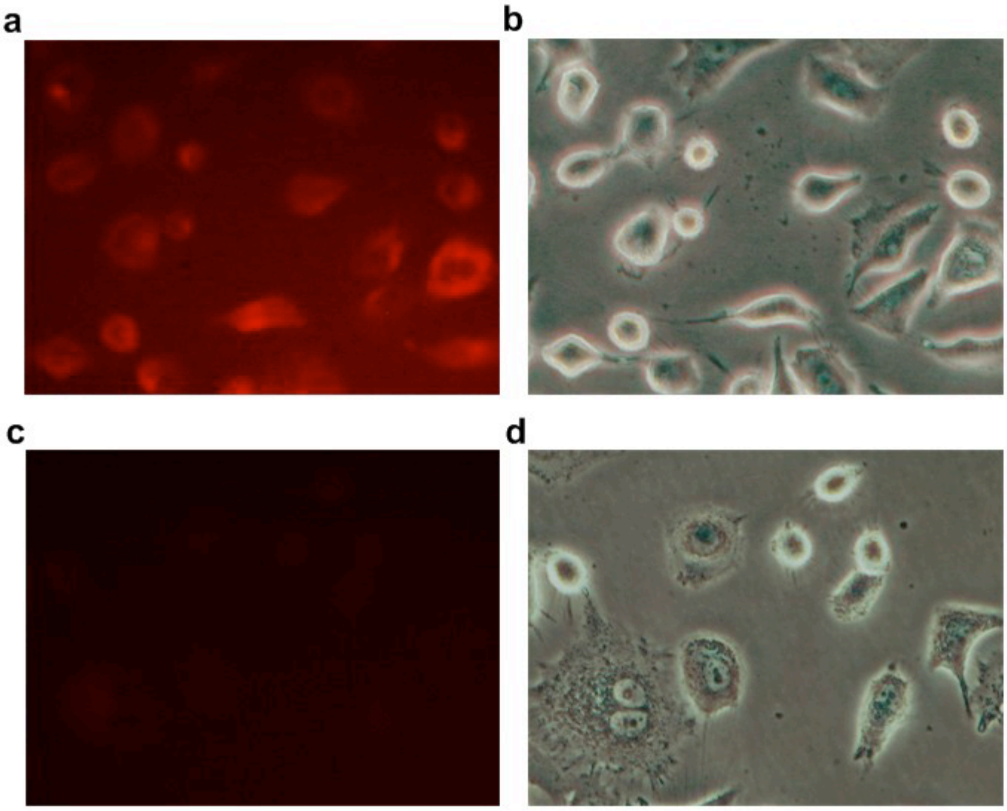

Figure S6. The permeability of NSC749235 (a, b) and NSC764638 (c, d) on A549 cells. A549 cells were treated with 5 uM NSC749235 or NSC764638 for 6 h, followed by washing three times with PBS. Cells were detected by fluorescence (a, c) and phase contrast microscope (b, d).

	DNA sequences	Extinction coefficients ($M^{-1}cm^{-1}$)
HTG22	5'-d[$AG_3(T_2AG_3)_3$]-3'	228500
c-myc (pu27)	5'-d[$TG_4AG_3TG_4AG_3TG_4A_2G_2$]-3'	279900
c-kit 1	5'-d[$G_3AG_3CGCTG_3AGGAG_3$]-3'	213200
Hairpin DNA	5'-TTGGCCAATGTTTGGCCAA-3'	178200

Table S1 DNA sequences and extinction coefficients used in this study.

Delta G (kJ/mol)	NSC749235		
DNA:drug ratio \ DNA	Hairpin	Human telomere (potassium)	Human telomere (sodium)
1:0	-31.79±1.1	-23.23±0.1	-15.45±0.2
1:1	-29.12±0.1	-24.00±0.4	-15.74±0.5
1:2	-30.91±0.5	-26.45±0.1	-16.56±1.0
1:4	-30.45±1.3	-29.95±0.1	-16.23±0.7
1:6	-31.03±0.4	-34.96±0.3	-16.60±0.3
1:10	-30.16±0.4	-35.31±1.8	-16.36±0.5

Table S2 Values of delta G of DNAs incubated in the presence of NSC749235 at various concentrations.

Delta G (kJ/mol)	NSC764638		
DNA:drug ratio \ DNA	Hairpin	Human telomere (potassium)	Human telomere (sodium)
1:0	-31.79±1.1	-23.23±0.1	-15.45±0.2
1:1	-30.51±1.2	-26.47±0.2	-16.97±1.1
1:2	-30.38±1.1	-31.64±1.4	-16.83±0.7
1:4	-30.48±0.2	-31.46±0.8	-16.70±0.6
1:6	-30.91±0.4	-33.57±0.7	-17.26±0.3
1:10	-31.19±0.9	-36.56±1.0	-18.79±0.9

Table S3 Values of delta G of DNAs incubated in the presence of NSC764638 at various concentrations.
